# Visual search patterns during exploration of naturalistic scenes are driven by saliency cues in individuals with cerebral visual impairment

**DOI:** 10.1038/s41598-024-53642-8

**Published:** 2024-02-06

**Authors:** Kerri Walter, Claire E. Manley, Peter J. Bex, Lotfi B. Merabet

**Affiliations:** 1 Translational Vision Lab, Department of Psychology, Northeastern University, Boston, MA USA; 2 The Laboratory for Visual Neuroplasticity, Department of Ophthalmology, Massachusetts Eye and Ear, Harvard Medical School, 20 Staniford Street, Boston, MA 02114 USA

**Keywords:** Human behaviour, Neurological disorders

## Abstract

We investigated the relative influence of image salience and image semantics during the visual search of naturalistic scenes, comparing performance in individuals with cerebral visual impairment (CVI) and controls with neurotypical development. Participants searched for a prompted target presented as either an image or text cue. Success rate and reaction time were collected, and gaze behavior was recorded with an eye tracker. A receiver operating characteristic (ROC) analysis compared the distribution of individual gaze landings based on predictions of image salience (using Graph-Based Visual Saliency) and image semantics (using Global Vectors for Word Representations combined with Linguistic Analysis of Semantic Salience) models. CVI participants were less likely and were slower in finding the target. Their visual search behavior was also associated with a larger visual search area and greater number of fixations. ROC scores were also lower in CVI compared to controls for both model predictions. Furthermore, search strategies in the CVI group were not affected by cue type, although search times and accuracy showed a significant correlation with verbal IQ scores for text-cued searches. These results suggest that visual search patterns in CVI are driven mainly by image salience and provide further characterization of higher-order processing deficits observed in this population.

## Introduction

### Eye movements and visual guidance

Given that the visual system acquires the highest resolution images at the fovea, visual information is gathered across multiple fixations when the center of gaze is focused on a sequence of singular points. While we can still see during these ballistic eye movements (saccades), sensitivity is greatly reduced as the fovea moves between points at high speed^1,2^. For this reason, we move our eyes frequently (2 to 4 times per second) to fixate on new locations in order to synthesize a coherent representation of the image or scene being explored^3^. Extensive research has been aimed at uncovering the factors that guide gaze behavior and what image features influence one fixation to the next, particularly with respect to viewing naturalistic scenes. While exact visual search patterns can certainly differ between individuals, it is possible to analyze gaze behavior to identify processing strategies that help explain why fixations land where and when they do. With this in mind, two major classes of information have been identified to explain what image features guide gaze behavior. The first, *image salience*, is predominately associated with bottom-up visual processing and is driven by low-level image features^4–7^. The second, *image semantics*, is associated with top-down processing and is highly influenced by prior knowledge and experience^8–15^.

### Cerebral visual impairment (CVI)

Early neurological damage to areas of the brain implicated with visual processing can lead to alterations in gaze behavior. One specific condition, referred to as cerebral visual impairment (CVI), is the most common cause of pediatric visual impairment in developed countries^16^. CVI has been defined as a brain-based visual disorder associated with damage and/or maldevelopment of retrochiasmal visual processing areas in the absence of major ocular disease^17,18^. The malfunctioning of key visual processing pathways is commonly associated with perinatal neurological injury and maldevelopment^19^. Examples include hypoxic-ischemic injury, trauma, and infection, as well as genetic and metabolic disorders^20,21^. While the profile of visual impairments in CVI is broad and complex (such as reduced visual acuity and contrast sensitivity, impaired visual field function, and ocular motor abnormalities^20,22^), higher-order visual perceptual deficits associated with impaired visuospatial processing and attention are also common^20,23–26^. This is particularly relevant in cases when visual acuity is at normal or near normal levels^27–29^, and thus, difficulties with higher-order processing may represent the most prominent clinical deficit in these individuals^30^. Finally, previous clinical accounts have also described that individuals with CVI often report difficulties with searching and extracting visual information in cluttered and complex visual scenes^31–35^. However, how these higher-order visual processing deficits influence eye movement guidance while exploring naturalistic scenes remains poorly understood.

### Image salience and image semantics

Image salience is calculated from the bottom-up, local conspicuity of features of an image (e.g., local change of luminance, color, contrast, edge orientation) such that gaze is guided to the most salient feature locations of a visual scene. A number of studies have provided experimental support for this notion, demonstrating that gaze is directed towards areas with high variation in image features that visually stand out or “pop out” from the background^4–7^. Based on this evidence, models have been developed that predict gaze behavior in which areas of a scene calculated as having high image salience have a correspondingly higher likelihood of fixation^36,37^.

In contrast, image semantics are computed from higher-level, top-down factors associated with prior knowledge and experience. Here, objects' meaning, context, and relationships within a scene influence gaze behavior. For example, an oven is often found in a kitchen but rarely in an office. Thus, based on prior knowledge, an oven in a kitchen scene would have high semantic salience and, accordingly, low semantic salience in an office regardless of its physical (i.e., image salience) properties. A number of studies have also shown how gaze is guided by image semantics based on the effect of environment^8–10,12,15^ and future actions^11,13,14^. From this evidence, corresponding models have been developed which successfully predict gaze behavior^12,38–44^.

**Figure 1:**
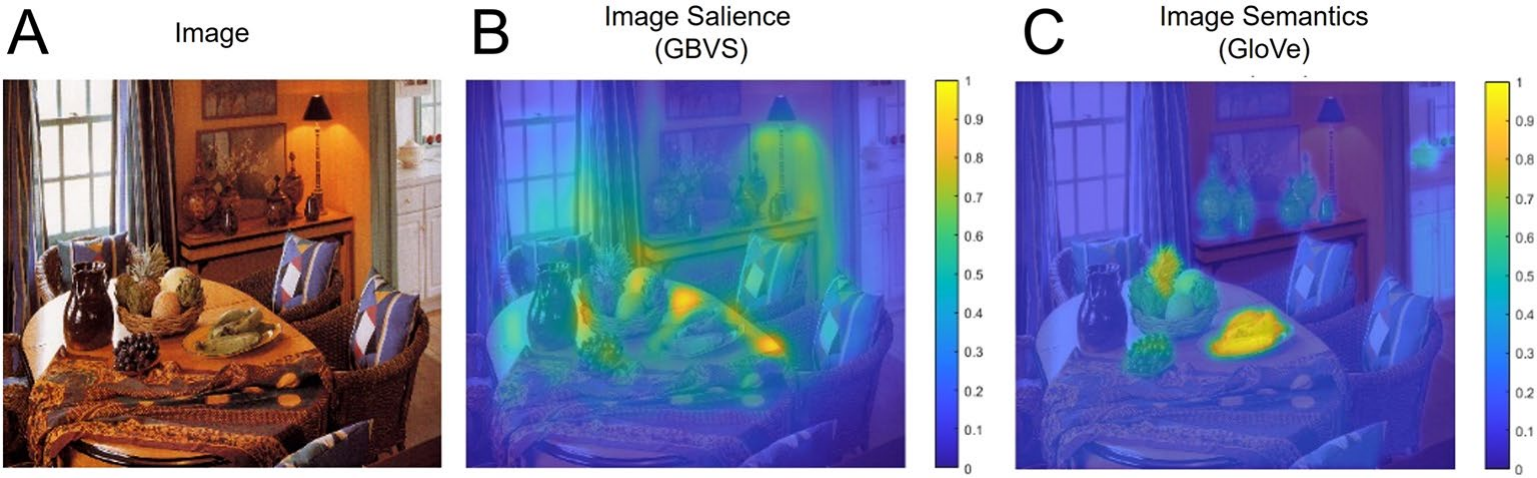
Examples of image analyses. (**A**) Original image of scene. (**B**) In the image salience condition (GBVS-based), the resulting heatmap identifies image features with high local contrast. (**C**) In the image semantics condition (GloVe-based), the resulting heatmap identifies the object corresponding to target word (in this example, “bananas”) as well as other objects (i.e. fruit) that have high semantic similarity. The scale shown is from 0 to 1, where 0 corresponds to an area unlikely to be fixated and 1 is an area that is likely to be fixated (according to the respective model predictions). Images shown are taken from the LabelMe database^74^, made publicly available to the research community and without restrictions. Images were created using Matlab Version 9.6 (https://www.mathworks.com/products/matlab.html).

Representative examples of image analysis based on these two major classes of information are shown in Fig. 1 (see also Sect. “Methods” for further details regarding visual image analysis).

### Present study aim

**Figure 2:**
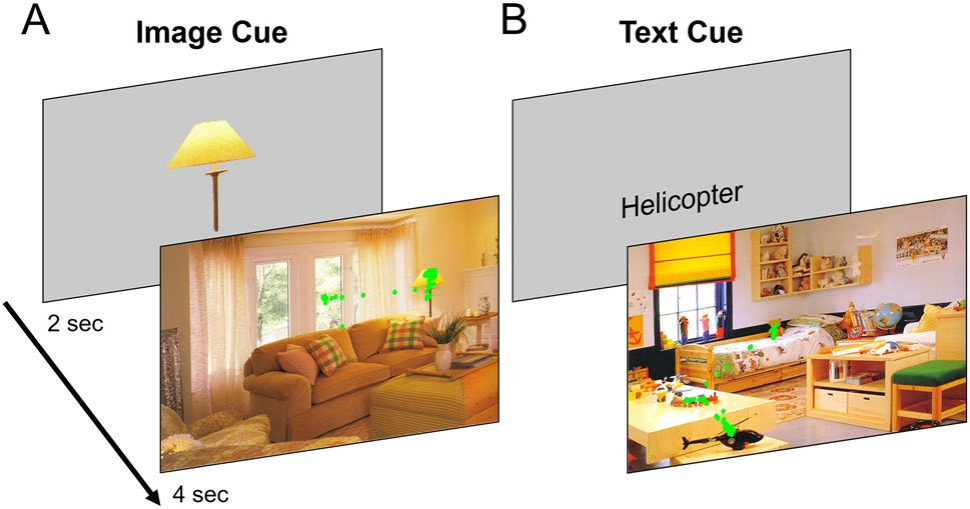
Experimental procedure. Participants were shown either an image cue or a text cue prompt for 2 s. The visual scene was then shown for 4 s and participants were instructed to locate and fixate on the target object until the end of the trial. Images shown are taken from the LabelMe databas^74^, made publicly available to the research community and without restrictions.

In this study, we compared the relative contribution of image salience and image semantics on visual guidance in relation to the exploration of naturalistic scenes in individuals with CVI compared to controls with neurotypical development. Given that higher-order visual perceptual deficits are commonly observed in CVI, we expected that individuals with this condition would show greater impairment (as compared to controls) in finding targets embedded in naturalistic scenes, and in particular, with respect to gaze behavior predicted by image semantics. Additionally, we examined the effect of the target cue on search behavior. Specifically, participants were asked to search for a predetermined target (prompted as either the target object presented in isolation or a text cue identifying the target object) followed by a naturalistic scene to be explored (see Fig. 2 for examples and Sect. “Methods” for further details). We used an eye tracker to record gaze behavior associated with the visual search. Our primary analysis focused on task performance based on success rate and reaction time. Secondary analyses investigated the extent of visual search area explored and number of fixations. To assess the predictive value of image salience and image semantics models, we deployed a graph-based visual saliency (GBVS^5^) model and a semantic saliency model^44^ incorporating Global Vectors for Word Representations (GloVe^45^) in conjunction with Linguistic Analysis of Semantic Salience (LASS^42^), respectively. The resultant predictions were based on the distribution of individual gaze landings captured for each visual scene. A receiver operating characteristic (ROC) analysis was carried out to compare the degree of correspondence between the distribution of individual gaze landings and image features predicted by the image salience and image semantics models.

Based on this experimental approach, a number of hypotheses could be generated. First, given that CVI is associated with early neurological injury, we surmised that visual search performance in individuals with this condition would be worse compared to controls with respect to our primary visual search outcomes (specifically, lower accuracy and longer reaction time). CVI participants would also show more difficulty searching for the targets characterized by our secondary analysis outcomes (including a larger overall visual search area and greater number of fixations). Regarding the effect of the target cue, we hypothesized that the image salience (GBVS-based) prediction would show greater agreement with gaze behavior patterns following visual object cues, while agreement with the image semantics (GloVe-based) prediction would be higher for gaze behavior patterns in response to text cues (as indexed by higher ROC score values). We also predicted that image semantics would be a worse predictor of overall gaze behavior in CVI participants due to their associated deficits with higher-order visual processing. Specifically, their gaze behavior would be more influenced by low-level features and accordingly, we expected that image salience would be a stronger predictor of gaze behavior in CVI in the context of exploring naturalistic visual scenes.

## Results

### Analysis of image complexity

Prior to our analysis of behavioral data, we confirmed that there were no significant associations between the number of objects (according to the total number of labeled objects, see Methods) or the complexity of the images (confirmed using the “entropy” function in Matlab, see Methods) [control image salience (R^2^ = 0.012, *p* = 0.327; R^2^ = 0.001, *p* = 0.761) and control image semantics (R^2^ = 0.023, *p* = 0.176; R^2^ = 5.1^e−5^, *p* = 0.950), CVI image salience (R^2^ = 0.009, *p* = 0.392; R^2^ = 0.042, *p* = 0.069), CVI image semantics (R^2^ = 0.027, *p* = 0.148; R^2^ = 0.010, *p* = 0.384) scores]. This analysis confirmed that the visual scenes explored were comparable across testing conditions and between both testing groups with respect to the number of objects and their image complexity.

### Behavioral outcomes

**Figure 3:**
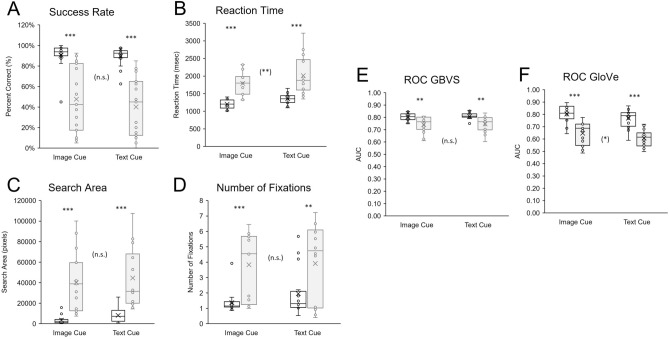
Behavioral results. Box and whisker plots comparing performance on the image cue and text cue conditions for controls (white) and CVI (gray) participants across all outcomes. (**A**) Success rate, (**B**) Search area, (**C**) Reaction time, (**D**) Number of fixations, ROC scores for the (**E**) image saliency (GBVS) and (**F**) image semantics (GloVe)-based models. Boxes represent upper and lower interquartile range; whiskers represent minima and maxima. Points correspond to outliers, and lines within boxes represent median values while the X symbol within boxes represent mean values. **p < 0.01, ***p < 0.001.

Regarding our primary behavioral outcomes, there was a significant effect of group regarding success rate (F(1,27) = 34.174, *p* < 0.001, η_p_^2^ = 0.559), no significant effect of cue (F(1,27) = 0.751, *p* = 0.394, η_p_^2^ = 0.072), and no significant interaction effect between group and cue on success rate (F(1,27) = 0.252, *p* = 0.620, η_p_^2^ = 0.009). Controls had a higher success rate than the CVI group for both the image cue condition (controls: 90.63% ± 12.99 SD, CVI: 47.67% ± 32.05 SD; t(18.241) = 4.832, *p* < 0.001, d = 1.779) and the text cue (controls: 89.69% ± 9.57 SD, CVI: 40.17% ± 29.16 SD; t(16.809) = 6.268, *p* < 0.001, d = 2.314) conditions (Fig. 3A). These findings suggest that CVI participants were less likely to find the target compared to controls.

For reaction time, there was a significant effect of group (F(1,27) = 34.629, *p* < 0.001, η_p_^2^ = 0.562) and cue (F(1,27) = 9.805, *p* = 0.004, η_p_^2^ = 0.266) and no significant interaction effect between group and cue (F(1,27) = 0.191, *p* = 0.665, η_p_^2^ = 0.007). Controls had a lower reaction time compared to CVI participants for both the image cue (controls: 1201.33 ms ± 131.55 SD, CVI: 1792.18 ms ± 332.84 SD; t(18.040) = − 6.421, *p* < 0.001, d = − 2.365) and text cue (controls: 1356.83 ms ± 149.62 SD, CVI: 2011.50 ms ± 544.33 SD; t(14.720) = − 4.358, *p* < 0.001, d = − 1.693) conditions (Fig. 3 B). These findings suggest that CVI participants were approximately 50% slower than controls in finding the target. Furthermore, overall, participants were approximately 13% faster in finding the target when presented with an image compared to text search cue.

**Figure 4:**
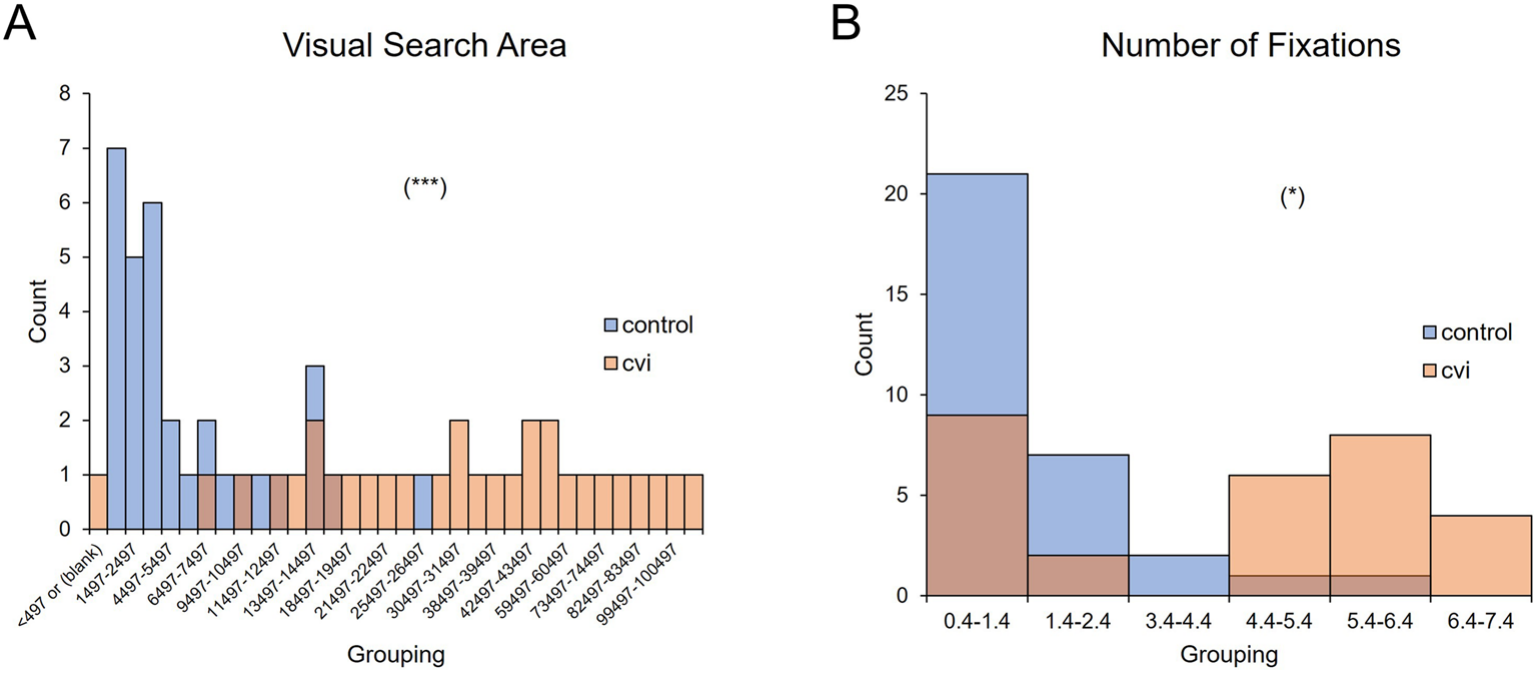
Distributions of visual search area and number of fixations between control and CVI groups. Both the distributions of (**A**) Visual search area and (**B**) Number of fixations were significantly different when comparing controls (blue) and CVI participants (orange). *p < 0.05, ***p < 0.001.

For the secondary outcomes, there was a significant effect of group on visual search area (F(1,27) = 25.303, *p* < 0.001, η_p_^2^ = 0.484), no significant effect of cue (F(1,27) = 3.909, *p* = 0.058, η_p_^2^ = 0.126), and no significant interaction between group and cue (F(1,27) = 0.420, *p* = 0.523, η_p_^2^ = 0.015). Controls had a smaller visual search area compared to the CVI group in both the image (controls: 3518.85 pixels ± 3959.75 SD, CVI: 40,096.96 pixels ± 29,190.94 SD; t(14.483) = − 4.812, *p* < 0.001, d = − 1.786) and text cue (controls: 8150.03 pixels ± 6711.03 SD, CVI: 44,614.20 pixels ± 29,312.69 SD; t(14.194) = − 4.551, *p* < 0.001, d = − 1.773) conditions (Fig. 3C). We further investigated whether visual search area was similarly distributed between the two experimental groups and found that the distributions were significantly different (U = 231.00, z = 4.388, *p* < 0.001). Visual inspection revealed that the distribution of visual search area was more tightly clustered in controls, while in CVI participants, visual search area was more broadly distributed (Fig. 4A).

There was also a significant effect of group on the number of fixations (F(1,27) = 13.031, *p* = 0.001, η_p_^2^ = 0.326), no significant effect of cue (F(1,27) = 0.786, *p* = 0.383, η_p_^2^ = 0.028), and no significant interaction between group and cue (F(1,27) = 1.730, *p* = 0.200, η_p_^2^ = 0.060). The control group made fewer fixations compared to CVI in both the image cue (controls: 1.37 fixations ± 0.71 SD, CVI: 3.83 fixations ± 2.19 SD; t(16.790) = − 4.155, *p* < 0.001, d = − 1.534) and the text cue (controls: 1.95 fixations ± 1.51 SD, CVI: 3.91 fixations ± 2.53 SD; t(20.575) = − 2.532, *p* = 0.002, d = − 0.958) conditions (Fig. 3D). We also investigated whether the number fixations made were similarly distributed between the two experimental groups and found that the distributions were significantly different (U = 176.500, z = 2.234, *p* = 0.024). Visual inspection revealed that controls generally made 1–2 fixations while CVI participants appeared to have a bimodal distribution (with peaks at 1–2 and 5 fixations, Fig. 4B).

### ROC analysis

There was a significant effect of group on image salience ROC scores (F(1,27) = 13.783, *p* < 0.001, η_p_^2^ = 0.338), no significant effect of cue (F(1,27) = 1.429 *p* = 0.242, η_p_^2^ = 0.050), and no significant interaction between group and cue (F(1,27) = 0.019, *p* = 0.891, η_p_^2^ = 0.001). Controls had higher image salience ROC scores compared to the CVI group for both the image (controls: 0.80 ± 0.03 SD, CVI: 0.74 ± 0.06 SD; t(19.993) = 3.401, *p* = 0.003, d = 1.248) and text cue (controls: 0.81 ± 0.03 SD, CVI: 0.75 ± 0.07 SD; t(17.233) = 3.284, *p* = 0.004, d = − 0.958) conditions (Fig. 3E). These results suggest that overall, gaze behavior was in closer agreement with image salience predictions for controls compared to CVI participants, and for both the image and text cue conditions.

There was a significant effect of group on image semantics ROC scores (F(1,27) = 43.532, *p* < 0.001, η_p_^2^ = 0.267), a significant effect of cue (F(1,27) = 5.052, *p* = 0.0332, η_p_^2^ = 0.267), with no significant interaction between group and cue (F(1,27) = 0.108, *p* = 0.745, η_p_^2^ = 0.004). For the semantic model prediction, controls had higher ROC scores compared to the CVI group for both the image (controls: 0.80 ± 0.08 SD, CVI: 0.65 ± 0.09 SD; t(27.005) = 5.144, *p* < 0.001, d = 1.862) and text (controls: 0.77 ± 0.08, CVI: 0.60 ± 0.07 SD; t(27.926) = 6.08, *p* < 0.001, d = 2.212) cue conditions (Fig. 3F). This suggests that overall, gaze behavior was in closer agreement with semantic prediction for controls compared to CVI participants. Furthermore, gaze behavior based on the image semantics prediction was more in agreement for the image compared to text cue across all participants.

Comparing ROC scores across the two models of gaze guidance revealed that there was a greater discrepancy between controls and CVI participants for the image semantics compared to the image salience prediction. We also examined ROC scores for salience and semantic guidance predictions as a function of fixation number. We found that in controls, the first fixations made (1–3) showed the highest ROC scores for both the image salience and image semantics predictions. By comparison, in the CVI group, ROC scores for both image salience and image semantics predictions were lower and steadier across fixations (see supplementary Fig. 1).

### Associations between verbal IQ and visual search performance in CVI

As an ancillary analysis, we explored whether verbal IQ scores in our CVI participants were associated with visual search performance based on our primary outcomes of interest. Specifically, we ran linear regression analyses between verbal IQ scores and success rate and reaction time independently, and for both the image and text cue tasks separately. We found that success rate was not significantly predicted by verbal IQ for the image cue condition (b = 0.233, t(12) = 0.826, *p* = 0.425), nor did it explain the variance in success rate (R^2^ = 0.054, F(1,12) = 0.682, *p* = 0.425). However, verbal IQ did significantly predict success rate in the text cue (b = 0.633, t(11) = 3.166, *p* = 0.009), and explained a significant proportion of the variance in success rate (R^2^ = 0.477, F(1,11) = 10.024, *p* = 0.009, see supplementary Fig. 2A). Similarly, we also found that reaction time was not significantly predicted by verbal IQ for the image cue condition (b = − 4.408, t(12) = − 1.495, *p* = 0.161), nor did it explain the variance in reaction time (R^2^ = 0.157, F(1,12) = 2.236, *p* = 0.161). However, verbal IQ did significantly predict reaction time in the text cue condition (b = − 9.887, t(11) = − 2.330, *p* = 0.040) and explained a significant proportion of the variance in reaction time (R^2^ = 0.331, F(1,11) = 5.431, *p* = 0.040, see supplementary Fig. 2B). Taken together, these results suggest that for our CVI participants, higher verbal IQ scores were associated with a higher success rate and faster reaction times in finding the target when presented as a text cue.

## Discussion

In this study, we investigated gaze behavior while participants searched for a predetermined target embedded in a naturalistic scene and compared performance in controls with neurotypical development and individuals with CVI; a neurodevelopmental disorder associated with early neurological damage and higher-order visual processing deficits. We also compared associations between the distribution of individual eye gaze patterns (i.e. gaze landings) and predictions of gaze behavior based on image salience (using GBVS) and image semantics (GloVe-based) models. Finally, we investigated the effect of the target cue on search performance, that is, when prompted as either the target object presented in isolation or as a text cue identifying the target object.

Compared to controls, we found that CVI participants were less likely to find and were slower in finding the target. Furthermore, visual search patterns in CVI were associated with a larger visual search area and greater number of fixations. Finally, comparing ROC scores across the two models of visual guidance revealed that there was a greater discrepancy between controls and CVI participants for the image semantics compared to the image salience predictions.

The observation of impaired visual search performance in CVI is consistent with previous work by our group^46,47^ as well as the clinical literature^31–35^. Thus, our results provide further objective evidence supporting clinical observations that individuals with CVI often have difficulties searching and finding an object, especially in complex and cluttered visual scenes. Our findings that the CVI participants also tended to explore over a larger visual search area and with a greater number of fixations further demonstrate that they not only took around 50% longer to find the target, but they also searched a greater proportion of the image and needed more fixations to do so (as opposed to simply taking a longer time within the same search area). This suggests that while CVI participants were actively searching the scene for the target, they were having more difficulty individuating the target and ended up scanning a larger proportion of the image. It is important to note that our image content analyses also confirmed that the visual scenes explored were comparable across testing conditions and between both groups with respect to number of objects and their image complexity. Thus, the observed differences between the control and CVI groups were not likely related to differences in the targets and visual scenes explored.

We also hypothesized that overall, the image salience prediction (GBVS) would be higher when participants were searching following an image cue given the affordance of color, orientation, and luminance properties of the target. In contrast, the semantic prediction (GloVe-based) would be higher for text-based cues, given that these cues did not specify the image properties of the target, and rather, would be more strongly influenced by prior knowledge. Interestingly, we found no significant effect of cue type for the image salience prediction, and a significantly higher ROC score for the image cue in the semantic prediction for controls (note that no statistically significant effect for the CVI group was observed). This meant that for controls, image salience was a stronger predictor of gaze behavior when searching for a target identified by a text cue. In other words, when the cue was presented as text (e.g. “desk”), participants fixated locations with high feature contrast, without knowing the composition or features of the specific desk they were searching for and thus not knowing whether the target had high or low salience relative to a background (e.g. a blue desk on a yellowish or bluish background). This may have forced participants to search the visual scene in a more serial manner, as they did not have access to the image feature cues provided in the image cue condition. While participants had previous knowledge of what desks look like and where they would likely appear^48,49^, they did not have any knowledge of the exact details of the particular desk they were searching for. This could have led to fixations being directed at any visually salient feature first (as visually salient features are known to capture gaze^36,37^, as a “default” strategy until the target was found. In other words, in the absence of image features to search for, gaze behavior our control participants were more likely guided by image salience rather than by image semantics. This is consistent with our previous work demonstrating that participants with neurotypical development search visual scenes in a manner that is more in line with image salience predictions when using an ROC based analysis^44,50^.

Given that higher-order visual processing deficits are often observed in individuals with CVI, we hypothesized that the predicted pattern of gaze landings from this group would be worse (i.e. lower associated ROC scores) for the image semantics (GloVe) model compared to controls, given that the image semantics are assumed to be driven by higher-order processing cues. Accordingly, we also expected image salience (GBVS) predictions would be worse, as previous work by our group has demonstrated that individuals with CVI used image salience cues significantly less compared to controls when identifying familiar objects^51^. In this study, we found that controls had higher ROC scores for both the image salience and image semantic predictions. Crucially, however, the discrepancy was greater for the image semantics prediction. This demonstrates that the CVI group were less reliant on image semantics guidance than control participants, consistent with possible deficits related to higher-order visual processing. Additionally, we found a significant cue effect with the semantic condition (but not for image salience), driven by the control group. This can be interpreted to mean that the CVI group was less affected by the distinction in cue type, as they searched scenes in a similar manner regardless of whether the target cue contained visual or semantic information. This may further suggest that the CVI group did not integrate cue information in the same way as controls, meaning they did not use the information provided in the cue to help search the scene. For example, to search for a red fire hydrant, they would not use the image salience cue provided by “color contrast” to search for red objects on non-red backgrounds or they would not use the semantic salience cue of “often on the sidewalk” (based on prior knowledge) to search near the road. Instead, they appeared to search the scene in a less systematic manner until the target was found. The reason for this discrepancy in gaze behavior in the CVI group is not entirely clear, but it may be related to impaired visual imagery priming, that in turn leads to a deficit in attentional guidance^52^. Interestingly, a bottom-up guided viewing strategy is believed to be more prominent in younger ages^53–55^. If neurological damage occurs early in life (as is the case with CVI), perhaps the bias towards using an image salience strategy predominates, and the tendency towards developing a more top-down (i.e. image semantics) strategy is delayed.

Relevant to this discussion, we also found a significant association between visual search performance and verbal IQ scores in our CVI participants. Specifically, individuals with higher verbal IQ showed a positive association with higher success rates and faster reaction times when the target was presented as a text cue (note a similar trend was also observed for the image cue task, though this association did not reach statistical significance). By extension, this finding suggests that language proficiency may be associated with visual search performance. Specifically, a deficit in semantic abilities may have an impact on the ability to find a target in a naturalistic visual scene. Early neurological damage and maldevelopment (as in the case of CVI) may impair an individual’s ability to form higher-order relationships between objects^56^. Thus, we could expect that individuals with CVI who have difficulties in projecting prior object knowledge onto their understanding of real-world settings would have search patterns less influenced by semantic features and ultimately, may have more difficulties finding that target in a complex naturalistic scene.

The neurophysiological substrates underlying these observed differences in gaze behavior in CVI remain the subject of on-going investigation (see^57^ for a further discussion on this topic). Using a semi-quantitative MRI-based rating scale, work by Tinelli and colleagues (2020) has shed light on potentially important associations between brain lesion severity and visual disorders in the specific case of CVI associated with CP due to PVL^58^. Relevant to the discussion here, this group reported that the presence of subcortical brain damage was highly associated with impaired fixations (as well as saccades) in relation to ocular motor functions^58^. Recent studies using diffusion-based imaging have highlighted potential differences in the structural integrity of key pathways implicated in higher-order visual processing in CVI compared to controls. For example, reduced white matter integrity has been reported along the inferior longitudinal fasciculus (ILF)^59^ and superior longitudinal fasciculus (SLF)^60^ corresponding the neuroanatomical correlates of the ventral (implicated with object identification) and dorsal (visuospatial processing) visual pathways, respectively ^61,62^. More recent work from our group has also provided evidence of reduced structural integrity of the inferior fronto-occipital fasciculus (IFOF)^63^, which is an important pathway implicated in selective visual attention^64,65^. Thus, it is possible that early damage and maldevelopment of these key visual processing pathways may contribute to specific aspects of the higher-order visual processing deficits observed in CVI. Future studies associating the location and extent of white matter compromise in relation to task performance are needed to better understand brain structural-behavioral relationships in CVI with respect to visual processing abilities.

Finally, a number of possible limitations should be considered. Most notable relates to the inclusion/exclusion criteria of this study that likely limited the clinical profile of the CVI participants enrolled. Specifically, CVI participants had to have sufficient visual acuity, intact visual field function, and ocular motor (i.e. fixation) abilities to allow for eye-tracking calibration and high-quality data capture. Furthermore, these subjects underwent age-appropriate neuropsychological (i.e., verbal IQ) testing to allow the exploration of putative associations between developmental factors and behavioral outcomes. Accordingly, these factors may have led to a potential selection bias that limits the overall generalizability of our results. Thus, caution should be considered when extrapolating our observations regarding the nature (as well as magnitude) of these higher-order visual processing deficits across the entire CVI population. Future studies should incorporate task design modifications that can accommodate a wider range of visual functioning as well as cognitive abilities. At the same time, it is important to recognize that individuals with CVI often present with other neurological and neurodevelopmental co-morbidities such as cerebral palsy (CP), Attention-Deficit/Hyperactivity Disorder (ADHD), dyslexia, and Autism Spectrum Disorder (ASD). Future studies should also consider comparing performance in these groups (i.e. not diagnosed with CVI) to help disentangle the nature of visual processing abilities in with respect to neurodevelopment (see recent study by^66^^,^^67^ for a review on this topic).

## Methods

### Study participants

Sixteen participants with neurotypical development aged between 14 and 27 years old (mean age: 18.75 years ± 3.47 SD) served as controls. Fifteen participants previously diagnosed with CVI and aged between 8 and 23 years old (mean age: 15.73 years ± 5.09 SD) served as a comparative group. Comparing controls and CVI participants with respect to age revealed no statistically significant difference (t(24.526) = 1.915, *p* = 0.067, d = 0.697).

All CVI participants were previously diagnosed prior to participating in this study by eyecare professionals with extensive clinical experience working with this population (see^68^ for similar criteria regarding the diagnosis of CVI). Briefly, the diagnosis was based on a directed and objective assessment of visual functions (including visual acuity, contrast, visual field perimetry, color, and ocular motor functions), functional vision assessment (use of structured questionnaires, surveys, and activities^69–71^, a comprehensive refraction and ocular examination, as well as an integrated review of medical history and available neuroimaging and electrophysiology records (see also^20,72,73^ for similar criteria and protocol). Causes of CVI were diverse and included hypoxic-ischemic injury related to prematurity and complications occurring at childbirth, periventricular leukomalacia (PVL), as well as genetic and metabolic disorders. Five CVI participants were born prematurely (less than 37 weeks gestation). Associated neurodevelopmental comorbidities included cerebral palsy (CP). Best corrected binocular visual acuity ranged from 20/15 to 20/70 Snellen (or − 0.12 to 0.54 logMAR equivalent). All the CVI participants in this study cohort were categorized as having “functionally useful vision and who can work at or near the expected academic level for their age group” (“category 3”) based on previously defined functional criteria^17^. Exclusion criteria included any evidence of oculomotor apraxia (i.e. failure of saccadic initiation), intraocular pathology (other than mild optic atrophy), uncorrected strabismus, as well as hemianopia or a visual field deficit corresponding to the area of testing (see supplementary Table 1 for complete demographic details).

Language abilities in the CVI cohort were also collected based on available clinical data. Specifically, verbal IQ was assessed using subtests from the Wechsler Intelligence Scale for Children (WISC IV) and Adults (WAIS IV), 4th Edition (Digit Span, Similarities, and Vocabulary subtests of WISC IV and Digit Span, Similarities, Vocabulary, and Information subtests of WAIS IV) to obtain an index of verbal comprehension. The mean score for the CVI participants was 93.00 ± 31.06 SD (range of 44 to 148).

Control participants had normal or corrected-to-normal visual acuity and no previous history of any ophthalmic (e.g. strabismus, amblyopia) or neurodevelopmental (e.g. attention deficit hyperactivity disorder) conditions.

All study participants had visual acuities, intact visual field function within the area corresponding to the stimulus presentation, as well as fixation and binocular ocular motor functioning sufficient for the purposes of completing the task requirements and eye tracking calibration (see below).

Prior to data collection, written informed consent was obtained from all participants and a parent/legal guardian (in the case of a minor). The study was approved by the Investigative Review Board at the Massachusetts Eye and Ear in Boston, MA, USA, and carried out in accordance with the Code of Ethics of the World Medical Association (Declaration of Helsinki) for experiments involving humans.

### Visual image selection and salience analysis

Eighty images (40 indoor, 40 outdoor scenes) were sourced from the LabelMe image database^74^. The LabelMe image database is an opensource tool for labeling objects within a naturalistic visual scene. Images chosen had between 20 and 114 labeled objects (mean = 46.413 objects ± 21.704 SD) and were of similar complexity (see Results section for further analysis confirmation). Prior to conducting the experiment, pilot testing was completed to confirm that the presentation time chosen was appropriate for all participants and for the number of images viewed (i.e. total test time). We manually reduced the noise found in the LabelMe database according to the following set of criteria^42^. First, we removed descriptor words, removed test/duplicate/nonsense labels, corrected spelling errors, and translated non-English labels. Second, because we used GloVe as the basis for our image semantics predictor model (see below), and because GloVe does not handle more than one word at a time, we reduced any multi-words to a single word, and made sure all words existed correctly in the GloVe semantic space. For this purpose, we manually edited labels by finding suitable single-word replacements (e.g. “license plate” became “numberplate”) and combined valid words (e.g. “street light” became “streetlight”). If we were unsure of a suitable replacement, we consulted MATLAB’s “vec2word” function to find the closest possible word between the multi-words (e.g. “trash bin” became “bin”).

We utilized GBVS as our image salience model. The GBVS model is readily available as an open-source toolbox and has demonstrated strong reliability and success in predicting human fixations based on low-level features associated with bottom-up processing. GBVS generates three individual feature maps based on color, orientation, and intensity, and the average of these maps represents a heatmap of the most salient image features. The GBVS model also factors in a general center bias, where it predicts the center of the image to be fixated more frequently than the edges due to the nature of eye movements during visual search^3,7,75–81^. The generated heatmap ranges in values from 0 to 1, where 0 represents areas with low image salience and 1 represents areas with high image salience. High image salience is categorized by areas of a scene that differ starkly from their surroundings in one of the three mentioned feature categories. For example, high color salience would be an orange traffic cone on a gray road. High orientation salience would be the strong, straight line of a tree trunk against an empty sky. High intensity salience would be a bright light in a dimly lit room. We applied gaussian blur equal to the estimated pixel error reported by the manufacturer of the eye tracker used (Tobii 4C, see below for further details) to soften boundaries and minimize errors that might occur from slight deviations in reported gaze location (see Fig. 1B for a representative example).

For our image semantics prediction, we utilized the GloVe model in conjunction with LASS. The GloVe model is one of the latest and regularly updated language models available and we have previously modified the LASS model to incorporate GloVe specifically for visual search tasks^44^. Specifically, we used GloVe to quantify image semantics by measuring how near two-word labels are in a defined “semantic space”. This semantic space is created by categorizing words across feature dimensions and placing them in a three-dimensional “web” of similarity. For example, all animal related words would cluster together, and all reptile related words would cluster within that cluster. In this way, the distance between two words represents the similarity between them (i.e. “frog” will be more related to “horse” than it would be to “airplane”). This similarity is quantified as the cosine distance between two words, where 0 = not similar and 1 = identical. This allows similarity values to be assigned to each object within a scene, where the comparison word is always the target object in the image. To spatially assign these values across the scene, we used LASS which is a method of generating context labels (i.e. the word used to compare all scene objects, in this case, the target object), calculating the semantic similarity scores (using GloVe), and embedding these scores within object masks defined by LabelMe. The result is a heatmap where all objects are scored based on their similarity to the target object (where the area within unrelated objects will be close to 0 and the area within the target object will equal 1). For example, in a scene where the target object was “boots”, the area labeled “floor” would have higher semantic salience than the area labeled “desk”, because boots are more often located on the floor compared to a desk. As with the image salience maps, we applied the same gaussian blur (see Fig. 1C for a representative example).

### Testing procedure

Participants were seated comfortably in a quiet room, 60 cm in front of a 17″ LED monitor (Alienware laptop computer, 1080p; 1920 × 1080 resolution). Eye movement patterns during visual search (i.e. X, Y coordinate positions of gaze) were captured under binocular viewing conditions using a Tobii 4 C Eye Tracker system (90 Hz sampling frequency, Tobii Technology AB, Stockholm, Sweden) mounted on the lower portion of the monitor. Participants were reminded to maintain their gaze on the monitor during testing but were otherwise able to move their head freely. Prior to each experiment, eye tracking calibration was performed on each participant (Tobii Eye Tracking Software, v 2.9 calibration protocol) which took less than one minute to complete. The process included a 7-point calibration task (screen positions: top-left, top-center, top-right, bottom-left, bottom-center, bottom-right, and center-center) followed by a 9-point post calibration verification (i.e., the same 7 calibration points plus center-left and center-right positions). Accuracy criterion was defined by gaze fixation falling within a 2.25 arc degree radius around each of the 9 screen positions and confirmed by visual inspection prior to data collection (testing procedure description taken from previous studies by our group^46,47,51^).

Participants were shown 2 blocks of 40 images. For testing, participants received one block where all targets were presented as image cues, and one block presented as text cues. The order of the conditions presented was counterbalanced across participants. Participants were shown a target for 2 s (either as an object image or text cue), followed by the visual scene to be explored for 4 s (see Fig. 2). Participants were instructed to search for the target object within the scene, and to maintain their gaze on the object once it was located and until the end of the trial. In order to balance the design, two task variations were used: (1) Text A, Image B and (2) Text B, Image A. All targets that were text cues in one version were images in the opposite version (e.g. “fire hydrant” would be a text cue for half of the participants and an image cue for the other half, for the same search scene).

### Behavioral outcomes and statistical analyses

The primary visual search performance outcomes based on gaze behavior were success rate (measured by the percentage of trials participants were able to successfully find and fixate on the target object) and reaction time (measured as the time in milliseconds participants took to locate and fixate on the target object from the beginning of the scene presentation). The time period during cue presentation (i.e. when the object image or text cue was presented) was not included in the reaction time measurement.

Secondary visual search outcomes were visual search area and number of fixations. We measured the approximate area that participants searched using a kernel density analysis. We used the Matlab function “ksdensity” to plot the contours containing the gaze data. “ksdensity” returns a probability density estimate based on a normal kernel function for all sample data. Essentially, a 3D map is plotted where the peaks of the map correspond to higher density areas of gaze points. We then converted these 3D maps into 2D polygons, where the polygon traces the boundary of the plotted contours, and this area corresponds to the search area. To detect and measure the number of fixations, we used the function “NonParaFixLab”^82^. “NonParaFixLab” calculates the optimum speed and duration thresholds for a given trial and evaluates each gaze point according to those criteria. When a gaze point surpasses both the speed and duration thresholds determined for a given trial, that point and following qualifying points are classified as belonging to a single fixation. 

We used an ROC analysis to quantify the predictive power of the image salience (GBVS) and image semantics (GloVe-based) models. An ROC curve is created by measuring the number of hits, correct rejections, misses, and false alarms that occur at increasing salience levels across the heatmap. For example, when testing at level 0.5, only areas of the heatmap with a value of 0.5 or lower are considered as correctly predicted. Any gaze point that falls in areas of 0.5 or lower are considered hits, and any areas above 0.5 without gaze points are considered correct rejections. Similarly, any areas predicted that do not have gaze points are scored as misses, and points falling on unpredicted areas are considered as false alarms. From this, we can calculate the true and false positive rates, where the true positives rate equals true positives/(true positives + false negatives), and the false positive rate equals 1 − (true negatives/(true negatives + false positives)). We repeated this at 100 levels increasing from 0 to 1, where the resulting false positives are plotted on the X axis and true positives are plotted on the Y axis, to generate an ROC curve. We used the Matlab function “AUC_Judd”^83^ to calculate the ROC curves and the area under the curve (AUC; otherwise referred to as the ROC score). The higher the ROC score (AUC value), the higher the predictive power of the model following a scale from 0 to 1. An ROC score of 1 means that that the subject’s gaze corresponded to exactly where the model predicted, while an ROC score value of 0.5 means the model predicted no better than chance. An ROC score value of 0 means that gaze points fell entirely outside areas of the model prediction.

All statistical analyses were carried out using SPSS Statistics package (version 28; IBM, Armonk, NY). To evaluate differences between the CVI and control groups, as well as the effect of the target cue (object image compared to text cue) on search behavior, we performed separate repeated-measures analyses of variance (ANOVA) for all outcomes of interest (success rate, reaction time, visual search area, number of fixations, and ROC scores) with “group” as the between-subjects factor and “cue” as the within-subjects factor. Independent samples t-tests were performed for each cue separately in the case of significant group effects to confirm directionality. Paired-sample t-tests were performed for both groups separately where there were significant cue effects. Mann–Whitney U tests were conducted on data regarding visual search area and number of fixations to investigate whether these outcomes were similarly distributed between the CVI and control groups. As an ancillary analysis, we examined if success rates and reaction times were associated with verbal IQ scores in CVI participants. For this purpose, linear regression analyses between both the image cue and text cue conditions were performed separately. Effect sizes are reported as partial eta squared. One CVI participant was only able to complete half of the experiment (image cue only). No data were omitted from the analysis.

## Supplementary Information

Supplementary Information.

## Data Availability

The datasets generated during and/or analyzed during the current study are available from the corresponding author upon reasonable request and contingent to IRB approval.
