# Author Correction: Visual search patterns during exploration of naturalistic scenes are driven by saliency cues in individuals with cerebral visual impairment

**DOI:** 10.1038/s41598-024-70965-8

**Published:** 2024-09-09

**Authors:** Kerri Walter, Claire E. Manley, Peter J. Bex, Lotfi B. Merabet

**Affiliations:** 1 Translational Vision Lab, Department of Psychology, Northeastern University, Boston, MA USA; 2 The Laboratory for Visual Neuroplasticity, Department of Ophthalmology, Massachusetts Eye and Ear, Harvard Medical School, 20 Staniford Street, Boston, MA 02114 USA

Correction to: *Scientific Reports* 10.1038/s41598-024-53642-8, published online 06 February 2024

The original version of this Article contained errors in the Results, under the subheading ‘ROC analysis’, where incorrect values were stated.

As a result,

“For the semantic model prediction, controls had higher ROC scores compared to the CVI group for both the image (controls: 1201.33 ms ± 131.55 SD, CVI: 1792.18 ms ± 332.84 SD; t(27.005) = 5.144, p < 0.001, d = 1.862) and text (controls: 1356.83 ms ± 149.62 SD, CVI: 2011.50 ms ± 544.33 SD; t(27.926) = 6.08, p < 0.001, d = 2.212) cue conditions (Fig. 3F).”

now reads:

“For the semantic model prediction, controls had higher ROC scores compared to the CVI group for both the image (controls: 0.80 ± 0.08 SD, CVI: 0.65 ± 0.09 SD; t(27.005)  = 5.144, *p* < 0.001, d = 1.862) and text (controls: 0.77 ± 0.08, CVI: 0.60 ± 0.07 SD; t(27.926) = 6.08, *p* < 0.001, d = 2.212) cue conditions (Fig. 3F).”

The original Article has been corrected.

