# Supplementary material for: Visual search patterns during exploration of naturalistic scenes are driven by saliency cues in individuals with cerebral visual impairment
Source: Sci Rep. 2024 Feb 6;14:3074. doi: 10.1038/s41598-024-53642-8 (PMC10847433; doi:10.1038/s41598-024-53642-8)
Supplement: Supplementary file 1 — Supplementary Information. [file 41598_2024_53642_MOESM1_ESM.pdf]

Supplementary Materials

Supplementary Table 1. CVI Participant Demographics

Parcipant ID	Sex	Age (years)	Verbal IQ	Etiology; Comorbidities	Preterm/ Term	Binocular Visual Acuity (Snellen)	Binocular Visual Acuity (LogMAR)	Functional Classification
1	female	8	48	seizure disorder	term	20/20	0	3
2	female	14	73	in-utero stroke; CP	preterm	20/25	0.1	3
3	female	22	44	ischemic hypoxic ischemia	preterm	20/40	0.3	3
4	female	20	114	meningitis, infarct	term	20/40	0.3	3
5	female	15	63	seizure disorder	term	20/40	0.3	3
6	female	12	114	perinatal head injury, hypoglycimia, anoxia	term	20/20	0	3
7	female	20	148	birth complication	term	20/15	-0.12	3
8	female	23	135	PVL; CP	preterm	20/15	-0.12	3
9	female	23	100	decreased palcental perfusion; global developmental delay	term	20/70	0.54	3
10	male	18	120	genetic disorder	term	20/20	0	3
11	male	10	91	hypoxia, PVL	preterm	20/20	0	3
12	male	16	na	unspecified; developmental delay	term	20/25	0.1	3
13	male	15	94	PVL; CP	term	20/20	0	3
14	male	10	79	cystic PVL; CP	preterm	20/30	0.17	3
15	male	10	80	delayed myelination; myopathy	preterm	20/20	0	3

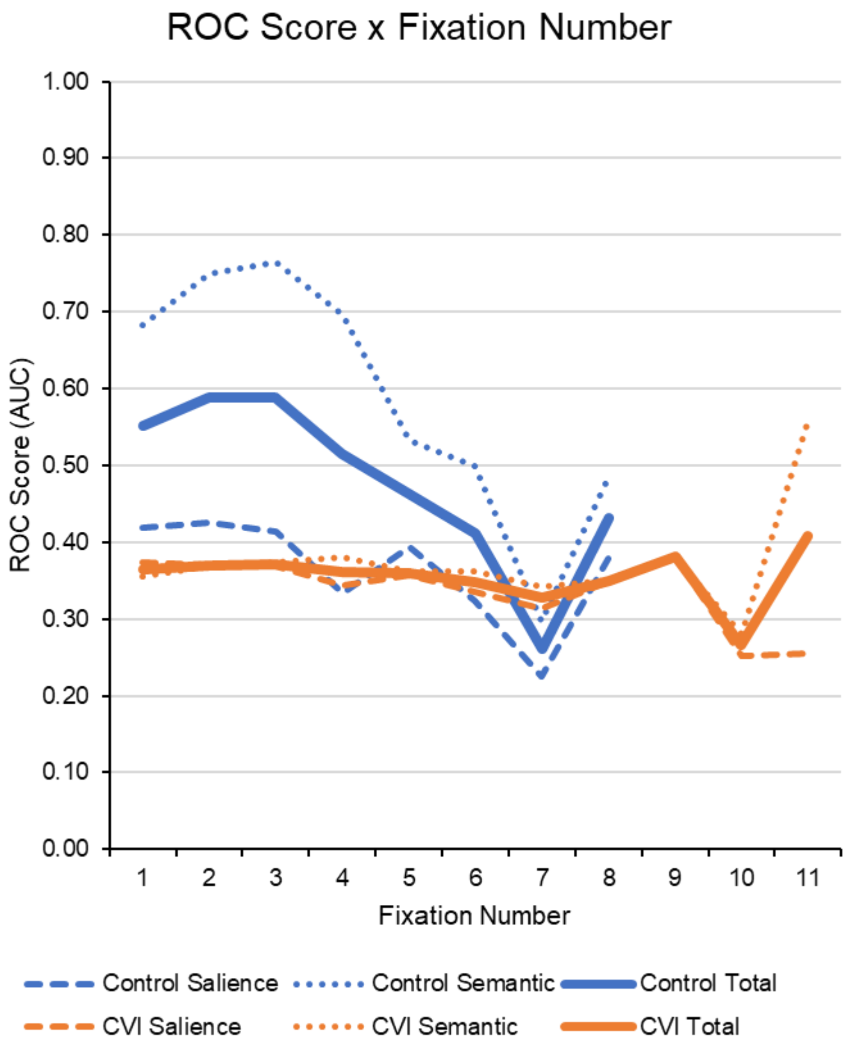

Supplementary Figure 1. Salience (ROC) Scores Across Number of Fixations. In controls (blue lines), the first fixations made (1 to 3) showed the highest ROC scores for both the image salience and image semantics predictions (note higher ROC score for image semantics). In the CVI group (orange lines), ROC scores for both the image salience and image semantics predictions were similar and steadier across fixations (as well as generally lower compared to controls).

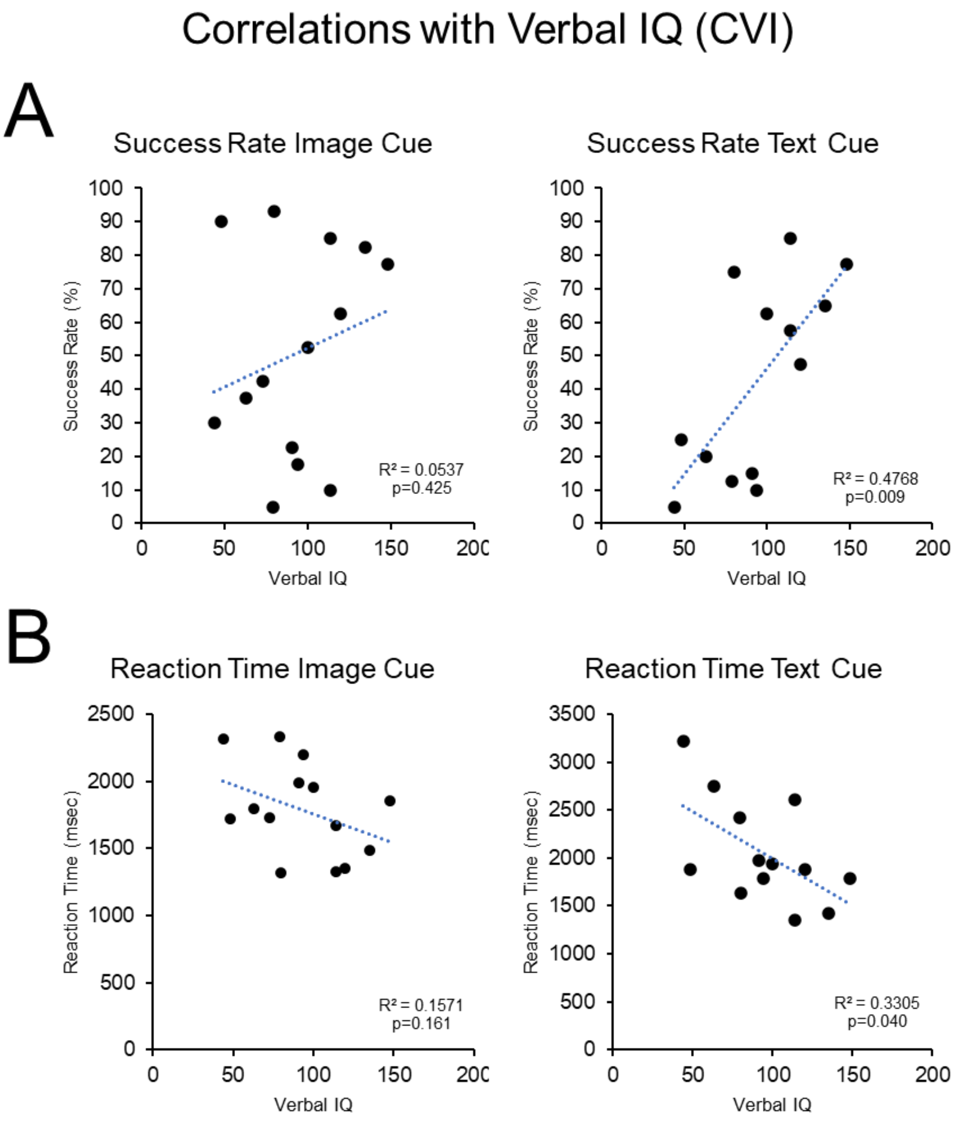

Supplementary Figure 2. Linear regressions in CVI participants with respect to visual search performance and verbal IQ. A) Success rate was not significantly predicted by verbal IQ in the image cue condition (left panel) but was significantly predicted by verbal IQ in the text cue condition (right panel). B) Reaction time was not significantly predicted by verbal IQ in the image cue condition (left panel) but was significantly predicted by verbal IQ in the text cue condition (right panel).
